# Benefits of Implantable Cardioverter–Defibrillator for Secondary Prevention in Patients With Organic Heart Disease

**DOI:** 10.1111/anec.70131

**Published:** 2025-12-10

**Authors:** Rie Akagawa, Sou Otsuki, Minori Sakurazawa, Atsushi Kato, Hironori Furuse, Naomasa Suzuki, Yasuhiro Ikami, Yuki Hasegawa, Masaomi Chinushi, Takayuki Inomata

**Affiliations:** ^1^ Niigata University Graduate School of Medical and Dental Sciences Niigata Japan; ^2^ Niigata University School of Medicine Niigata Japan

**Keywords:** appropriate ICD therapy, benefit, cardiac resynchronization therapy device with a defibrillator (CRT‐D), elderly, implantable cardioverter–defibrillator (ICD), secondary prevention, ventricular fibrillation

## Abstract

**Background:**

Implantable cardioverter‐defibrillators (ICD) are first‐line treatment to prevent sudden cardiac death due to recurrent ventricular tachycardia and fibrillation (VT/VF). However, some patients with organic heart disease (OHD) die without ever receiving appropriate ICD therapy. This study aimed to identify predictors of death without appropriate ICD therapy in patients with OHD who received ICD or cardiac resynchronization therapy with a defibrillator (CRT‐D) for secondary prevention.

**Methods:**

We analyzed consecutive patients who received ICD/CRT‐D for secondary prevention between 2000 and 2022. Patients without OHD or those alive without appropriate ICD therapy were excluded. The “no‐benefit group” included patients who died or developed severe disability without appropriate ICD therapy or those who died within 1 year after their first appropriate therapy. The “benefit group” included patients who survived > 1 year after appropriate therapy. Clinical characteristics were compared between the groups.

**Results:**

Of the 170 patients analyzed (median follow‐up: 9.1 years), 43 (25%) were classified into the no‐benefit group (30 died without appropriate therapy, 10 died within 1 year of first appropriate therapy, and 3 developed severe disability without appropriate therapy). Multivariate analyses identified age > 70 years and history of VF as independent predictors of “no benefit.” Among patients with VF aged ≥ 70 years, 71% were classified into the no‐benefit group.

**Conclusions:**

Although 75% of patients benefited from ICD therapy for secondary prevention, elderly patients with VF may gain limited benefits from ICD implantation.

## Graphical abstract

Three‐quarters of patients who underwent ICD/CRT‐D implantation for secondary prevention received appropriate ICD therapy and survived at least 1 year thereafter. In contrast, patients aged 70 or older and those with a history of ventricular fibrillation (VF) were less likely to benefit from secondary prevention with an ICD.

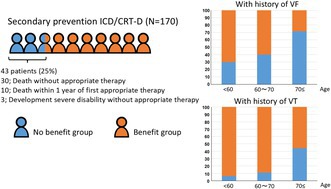

**Abbreviations**
ATP: antitachycardia pacing
CI: confidence intervals
CRT‐D: cardiac resynchronization therapy device with a defibrillator
ECG: electrocardiography
ICD: implantable cardioverter–defibrillator
VF: ventricular fibrillation
VT: ventricular tachycardia

## 1 Introduction

Multiple large‐scale randomized trials have demonstrated that implantable cardioverter–defibrillators (ICDs) are recommended for secondary prevention in patients who have survived life‐threatening ventricular arrhythmias due to their high risk of recurrence (Priori et al. 2015; Al‐Khatib et al. 2017; Connolly, Hallstrom, et al. 2000). In patients with organic heart disease (OHD), it is often difficult to avoid ICD implantation for ventricular tachycardia (VT) because current guidelines recommend it as a class IIa indication (Al‐Khatib et al. 2017; Nogami et al. 2021), even when catheter ablation and pharmacological treatments are effective for sustained VT.

However, the risk of complications from cardiac implantable electronic device therapy is not negligible—device‐related infections occur in approximately 4% of cases annually (Modi et al. 2023). Furthermore, a previous study reported a 14% incidence of perioperative complications, with risk factors including chronic kidney disease (CKD), advanced age, cardiac resynchronization therapy (CRT) implantation, and anticoagulant use (Ascoeta et al. 2016). Therefore, ICD implantation should be carefully considered, particularly in elderly patients with multiple comorbidities. Nonetheless, because guidelines firmly support ICD use for secondary prevention, it is ethically difficult to conduct prospective studies evaluating ICD efficacy in such cases. Although some patients ultimately experience no benefit from ICD therapy and are instead exposed to its risks, the characteristics of those who receive ICDs for secondary prevention but never experience appropriate therapy have not been fully described. Therefore, we focused on patients with OHD and retrospectively assessed which individuals may not have required an ICD for secondary prevention.

## 2 Materials and Methods

### 2.1 Study Population

This single‐center retrospective study included all consecutive patients who underwent ICD or CRT‐D implantation at our hospital between January 2000 and December 2019. We included patients with OHD and documented VT/VF. All patients received ICD/CRT‐D implantation for the standard indication.

**FIGURE 1:**
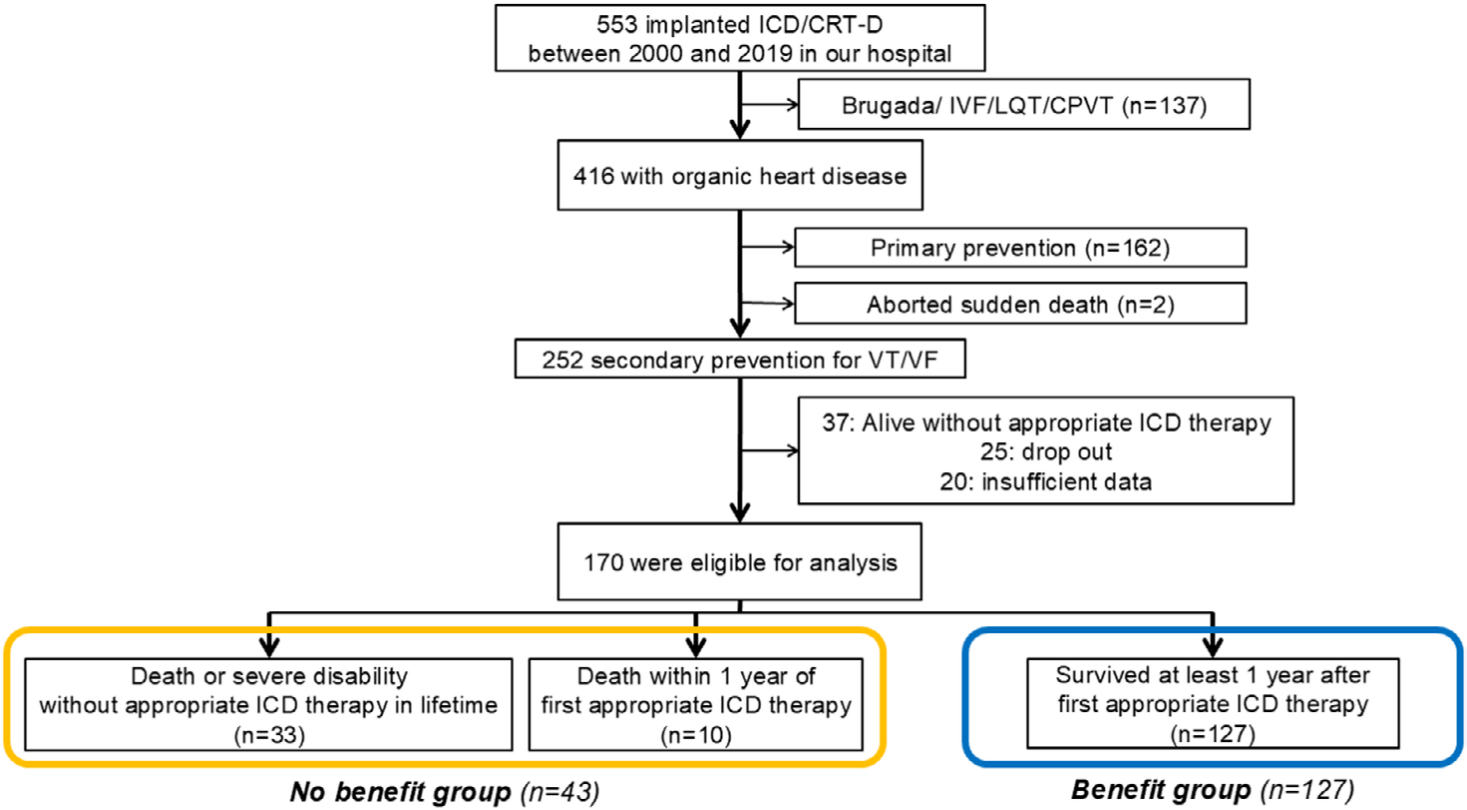
Enrollment criteria and flowchart of this study. CPVT, catecholaminergic polymorphic ventricular tachycardia; IVF, idiopathic ventricular fibrillation; LQT, long QT syndrome; VF, ventricular fibrillation; VT, ventricular tachycardia.

The primary aim of this study was to estimate the number of patients who died without appropriate ICD therapy after ICD/CRT‐D implantation. Furthermore, we sought to identify risk factors associated with death without appropriate ICD therapy during a patient's lifetime. Patients were categorized into two groups based on whether they died without appropriate ICD therapy or survived after appropriate ICD therapy—the benefit and non‐benefit groups. Considering that many ICD patients who were considered to be near the end of life receive appropriate ICD therapy within the year before death (Trussler et al. 2019) and that Japanese guidelines define ICD implantation for patients without consent or cooperation for ICD therapy because of a psychiatric disorder as class III and ICD implantation for patients with a predicted life expectancy ≤ 12 months as class III (Nogami et al. 2021), the following patients were classified into the non‐benefit group: (1) death without appropriate ICD therapy in lifetime (appropriate antitachycardia pacing [ATP] and/or appropriate ICD shock due to VF or sustained ventricular tachycardia), (2) inability to continue hospital visits due to severe disability without appropriate ICD therapy, and (3) death within 1 year of first appropriate ICD therapy. In contrast, we classified patients who survived at least 1 year after first appropriate ICD therapy into the benefit group. We excluded patients who were alive without appropriate ICD therapy, were lost to follow‐up, or had incomplete data on baseline characteristics (Figure 1).

This study was approved by the Ethics Committee of Niigata University and was conducted in accordance with the Declaration of Helsinki and the Ethical Guidelines for Medical and Health Research Involving Human Subjects. Details regarding the study's implementation, including the use of existing samples and data, were publicly disclosed, and patients were given the opportunity to opt out. Given the retrospective design, the Ethics Committee determined that these procedures fulfilled the requirements for informed consent and therefore granted a waiver for written consent. An opt‐out approach was used accordingly.

### 2.2 Clinical Data and Device Programming and Management

Collected clinical data included patient age, sex, type of underlying heart disease, prior catheter ablation for VT, medications, comorbidities, and pre‐implantation findings from electrocardiography (ECG) and echocardiography. VT/VF therapy settings were tailored by the attending physicians based on each patient's clinical condition. Among the 44 patients without a history of VT, VT detection zones were programmed in 22 cases; 15 of these patients subsequently received appropriate ICD therapy. All but two received therapy for rapid VT or VF exceeding 188 bpm. Several sequences of ATP were generally programmed to precede shock delivery within the VT zones.

### 2.3 Statistical Methods

Intergroup differences in clinical characteristics and course were determined using the unpaired *t*‐test for continuous variables and the chi‐square test for categorical variables. Quantitative data are presented as means ± standard deviations, medians and interquartile ranges, and ranges depending on data distribution and were compared using Student's *t*‐test for normally distributed data and the Mann–Whitney *U* test for nonparametric data. Logistic regression was used to evaluate the characteristics of the no‐benefit group, and the odds ratios together with their 95% confidence intervals (CIs) were reported. *p*‐values < 0.05 were used to denote statistical significance.

## 3 Results

### 3.1 Baseline Characteristics

In total, 553 patients underwent ICD/CRT‐D implantation during the study period (Figure 1). Among them, 252 with OHD underwent ICD implantation for secondary prevention of VT/VF. We excluded 37 patients who remained alive without appropriate ICD therapy, 25 patients who were lost to follow‐up, and 20 patients with incomplete medical records. Thus, 170 patients were included in the final analysis. Table 1 presents the baseline characteristics of the patients. At the time of ICD/CRT‐D implantation, the mean age of all patients was 61 ± 14 years, and 103 patients (75%) were male. Of the 170 patients, 51 (30%) had ischemic heart disease and 86 (51%) had nonischemic heart disease. The mean left ventricular ejection fraction for all patients was 47% ± 17%. Device implantation was indicated for VF in 44 patients (26%) and for VT in 126 patients (74%).

**TABLE 1:** Differences between patients with no‐benefit group versus benefit group (Among 170 patients).

	No‐benefit group (*n* = 43)	Benefit group (*n* = 127)	*p*
Age, years	66.0 ± 14.5	59.3 ± 13.7	**< 0.001**
Female, *N* (%)	11 (25%)	27 (21%)	0.53
BMI (kg/m^2^)	22.0 (19.5–25.6)	22.4 (20.4–25.3)	0.81
Underlying disease, *N* (%)			
Ischemic cardiomyopathy	22 (51%)	29 (22%)	**< 0.001**
Non‐ischemic cardiomyopathy	15 (34%)	71 (56%)	**0.022**
Valvular heart disease	2 (4%)	5 (3%)	1.0
Congenital heart disease	2 (4%)	7 (5%)	1.0
Others	2 (4%)	15 (12%)	0.24
CRT, *N* (%)	2 (4%)	13 (10%)	0.23
VF episode, *N* (%)	20 (46%)	24 (18%)	**0.001**
Comorbidities, *N* (%)			
Atrial fibrillation	14 (32%)	42 (33%)	0.85
Hypertension	20 (46%)	43 (33%)	0.15
Diabetes mellitus	17 (39%)	24 (18%)	**0.008**
Hyperlipidemia	14 (32%)	31 (24%)	0.32
eGFR < 60, *N* (%)	27 (62%)	40 (31%)	**< 0.001**
Anticoagulant, *N* (%)	27 (62%)	81 (63%)	1.0
A history of malignant tumor, *N* (%)	6 (13%)	6 (4%)	0.08
A history of PCI or CABG, *N* (%)	19 (44%)	23 (18%)	**0.001**
LV ejection fraction, (%)	47 ± 17	46 ± 17	0.34
QRS, (msec)	127 ± 34	129 ± 34	0.75
QTc, (msec)	451 ± 44	451 ± 48	0.33
Drugs for heart failure, *N* (%)			
ACE inhibitor/ARB	31 (72%)	87 (68%)	1.0
MRA	16 (37%)	42 (33%)	0.71
β‐blocker	29 (67%)	94 (74%)	0.43
Antiarrhythmic drugs class III, *N* (%)	25 (58%)	76 (59%)	0.86
Amiodarone	13 (30%)	29 (22%)	0.41
Sotalol	8 (18%)	40 (31%)	0.12

*Note:* Mean ± S.D. Bold indicates *p* < 0.05.

Abbreviations: ACEi, angiotensin‐converting enzyme inhibitor; ARB, angiotensin II receptor blocker; BMI, body mass index; CABG, coronary artery bypass grafting; CRT, cardiac resynchronization therapy; MRA, mineralocorticoid receptor antagonist; PCI, percutaneous coronary intervention.

Over a median follow‐up of 9.1 years (range, 5.2–13.1 years), 100 patients died, including 7 (4%) within 1 year of device implantation. Of the 100 patients, 30 patients died without ever receiving appropriate ICD therapy, and 10 died within 1 year after their first appropriate therapy. An additional three patients developed severe disability without receiving appropriate ICD therapy. These 43 patients were categorized into the no‐benefit group. In contrast, 137 patients (81%) received appropriate ICD therapy, including the 10 who died within 1 year post‐therapy; thus, 127 patients (75%) were classified into the benefit group (Figure 1). Lead failure was observed in 19 patients (11%), and 2 patients (1%) developed infections requiring device removal.

### 3.2 Predictors of the No‐Benefit Group

**FIGURE 2:**
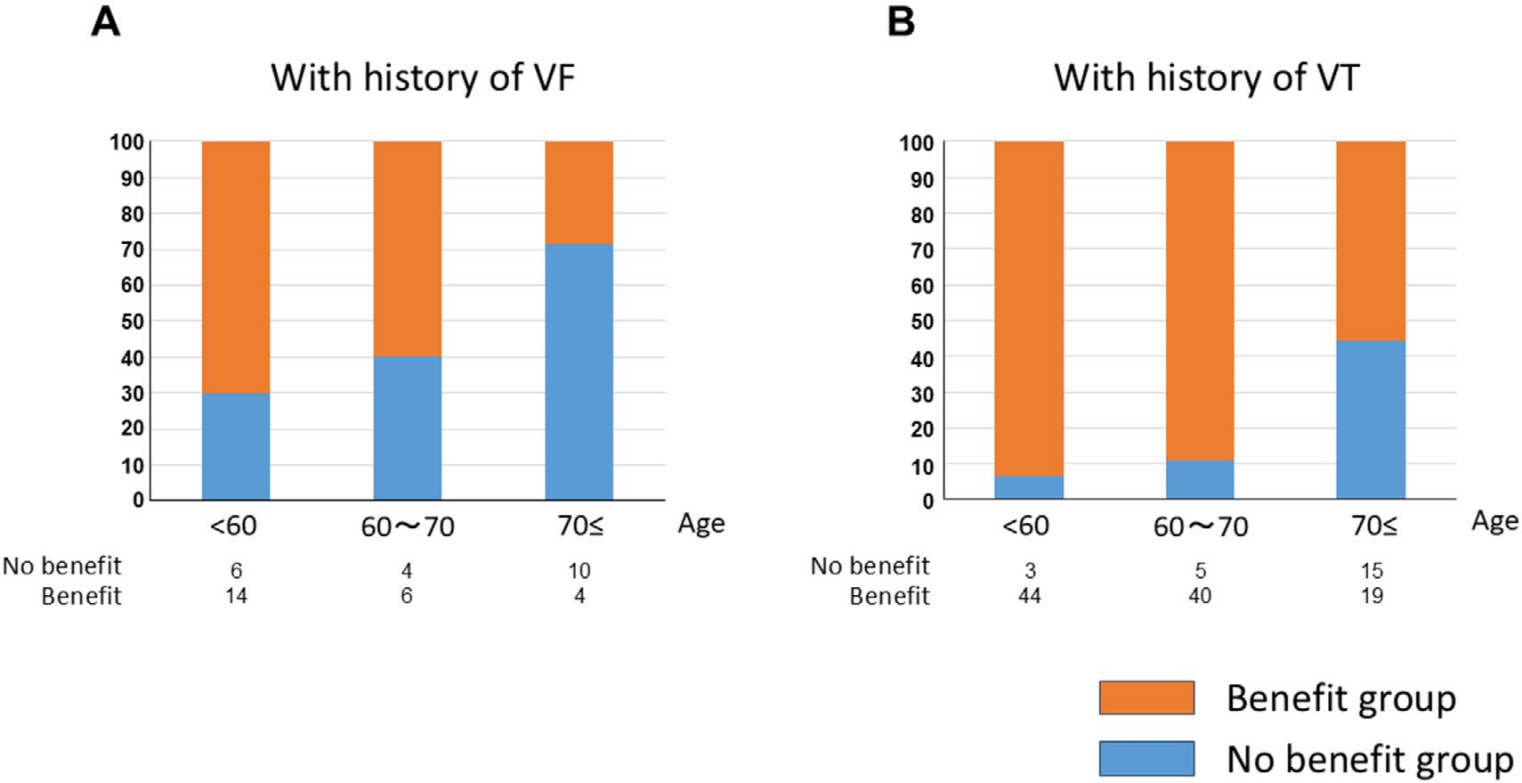
Frequency of outcomes at different ages in (A) patients with a history of VF and (B) those with a history of VT. The no‐benefit group was defined as (1) death without appropriate ICD therapy, (2) development of severe disability without appropriate ICD therapy, and (3) death within 1 year of first appropriate ICD therapy.

Table 1 outlines the clinical characteristics of patients in each group. Patients in the no‐benefit group were older than those in the benefit group. CKD, diabetes mellitus, a history of revascularization, and VF were significantly more prevalent in the no‐benefit group than in the benefit group (*p* < 0.01). Patients in the no‐benefit group had a higher prevalence of ischemic cardiomyopathy and a lower prevalence of nonischemic cardiomyopathy than those in the benefit group. Multivariate analyses identified age > 70 years (hazard ratio [HR], 4.45; 95% CI, 1.79–11.1; *p* = 0.001) and a history of VF (HR, 5.29; 95% CI, 2.14–13.1; *p* < 0.001) as independent predictors of being in the no‐benefit group (Table 2). Of the 14 patients aged ≥ 70 years with a history of VF, 10 (71%) were classified in the no‐benefit group (Figure 2).

**TABLE 2:** No‐benefit group risk factors at baseline (Among 170 patients).

	Univariate			Multivariate		
	OR	CI	*p*	OR	CI	*p*
Age > 70	6.28	2.95–13.4	**< 0.001**	4.45	1.79–11.1	**0.001**
Female	1.22	0.54–2.71	0.63			
BMI	0.97	0.89–1.07	0.60			
Underlying disease						
Ischemic cardiomyopathy	3.54	1.71–7.33	**< 0.001**	0.83	0.15–4.52	0.83
Non‐ischemic cardiomyopathy	0.42	0.21–0.87	**0.019**	0.70	0.22–2.26	0.55
Valvular heart disease	1.19	0.16–5.75	0.84			
Congenital heart disease	0.83	0.12–3.62	0.82			
Others	0.36	0.08–1.66	0.19			
CRT	0.43	0.09–1.98	0.28			
VF episode	3.73	1.77–7.87	**< 0.001**	5.29	2.14–13.1	**< 0.001**
Comorbidities						
Atrial fibrillation	0.98	0.47–2.04	0.95			
Hypertension	1.69	0.84–3.43	0.14			
Diabetes mellitus	2.81	1.38–5.97	**0.007**	1.74	0.63–4.80	0.29
Hyperlipidemia	1.49	0.70–3.18	0.30			
eGFR < 60	3.59	1.74–7.39	**< 0.001**	2.51	0.99–6.37	0.053
Anticoagulation	0.96	0.47–1.96	0.91			
A history of malignant tumor	3.27	0.99–10.7	0.051			
A history of PCI or CABG	3.58	1.69–7.60	**0.001**	1.69	0.41–7.04	0.47
LV ejection fraction	1.01	0.99–1.03	0.34			
QRS (/10 ms)	0.98	0.88–1.10	0.76			
QTc (/10 ms)	1.04	0.96–1.12	0.33			
Drug for heart failure						
ACEi/ARB	1.19	0.55–2.55	0.66			
MRA	1.20	0.58–2.46	0.62			
β‐blocker	0.73	0.34–1.54	0.41			
Antiarrhythmic drug class III	0.93	0.46–1.89	0.84			
Amiodarone	1.46	0.66–3.13	0.33			
Sotalol	0.49	0.19–1.12	0.094			

*Note:* Abbreviations are shown in Table 1.

### 3.3 Timing of Appropriate ICD Therapy, Death, and Generator Exchange

**FIGURE 3:**
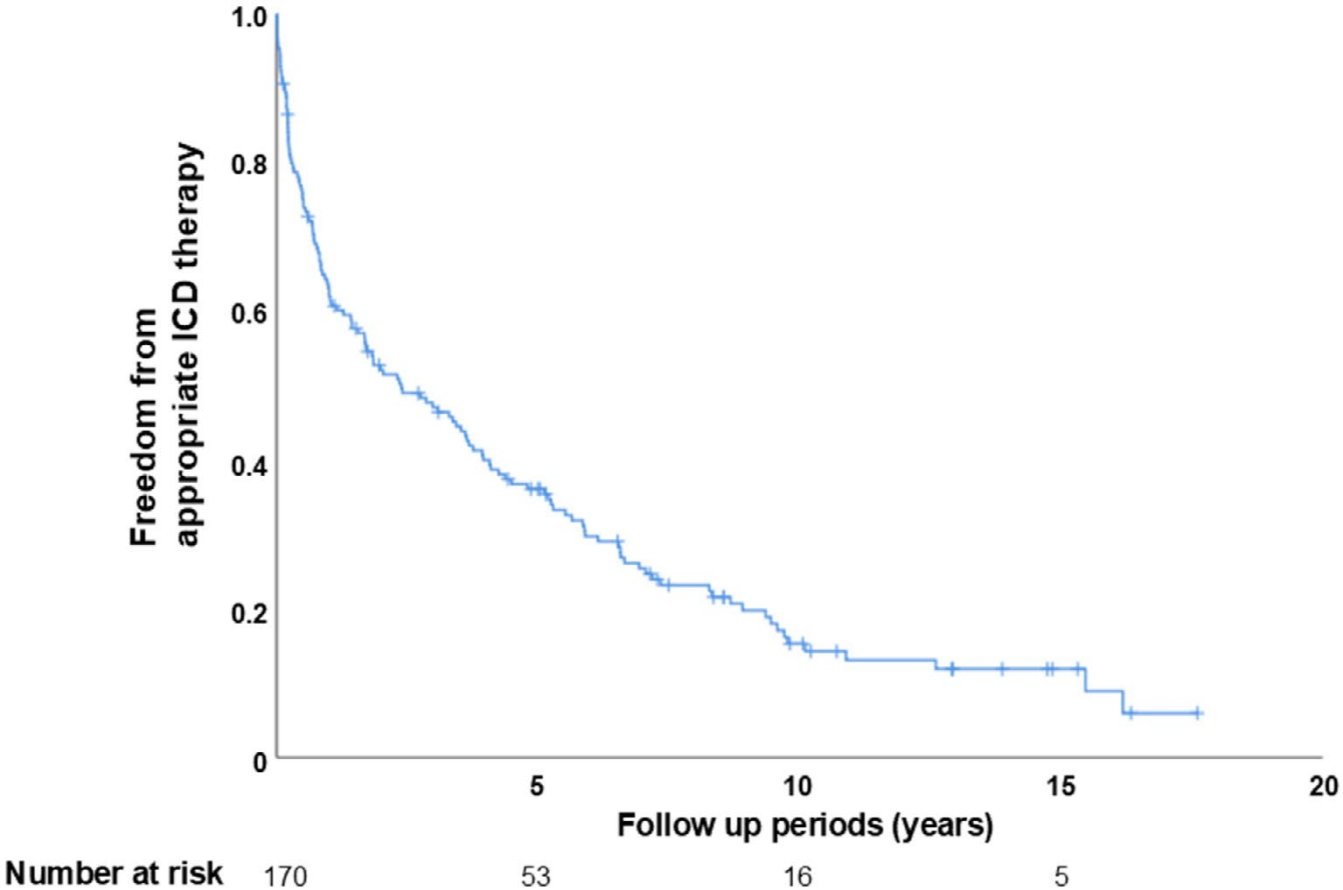
Occurrence of first‐ever ICD therapy over time.

The median interval from ICD/CRT‐D implantation to the first appropriate ICD therapy was 1.4 years (range: 0.3–4.4 years), with the longest being 16.2 years (Figure 3). The median duration from the first appropriate ICD therapy to death or transfer due to severe disability was 4.6 years (range: 1.8–9.0 years). During follow‐up, 111 patients underwent generator replacement. Among them, 39 had not received appropriate ICD therapy prior to the first generator exchange. Of these 39 patients, 23 (59%) experienced their first appropriate ICD therapy after the exchange, while 16 (41%) died without ever receiving appropriate therapy.

## 4 Discussion

### 4.1 Main Findings

The major results of this study were as follows: (1) 75% of patients who underwent ICD/CRT‐D implantation for secondary prevention received appropriate ICD therapy and survived at least 1 year thereafter, and (2) patients aged 70 or older and those with a history of ventricular fibrillation (VF) were less likely to benefit from secondary prevention with an ICD.

In the Antiarrhythmics Versus Implantable Defibrillators, Cardiac Arrest Study Hamburg, and Canadian Implantable Defibrillator Study trials, ICD demonstrated clear effectiveness for secondary prevention of fatal arrhythmias (Antiarrhythmics Versus Implantable Defibrillators (AVID) Investigators 1997; SKuck et al. 2000; Connolly, Gent, et al. 2000). However, these randomized controlled trials were conducted before 2000. A subsequent meta‐analysis that combined individual patient data from these three studies found that ICD therapy did not appear to provide a survival advantage in patients aged ≥ 75 years (Healey et al. 2007). In fact, some patients derive minimal benefit—some never receive appropriate ICD therapy during their lifetime or die within a year of implantation despite initial favorable prognoses. Moreover, ICDs are not without risks; patients may experience complications such as infections or lead‐related issues. Inappropriate ICD therapy has also been reported in approximately 9.4% of cases (Basu‐Ray et al. 2017).

In this study, a substantial proportion of patients—75%—derived benefit from ICD implantation. The median time from ICD/CRT‐D implantation to the first appropriate ICD therapy was relatively short at 1.4 years; however, the longest interval recorded was 16.2 years. A previous study noted that 3.3% of patients received their first appropriate ICD therapy more than 15 years post‐implantation (Schaer et al. 2016), indicating that while the risk of arrhythmic events decreases over time, it does not completely disappear. This finding is consistent with other studies that have followed patients over extended periods (Yap et al. 2014).

In this study, 59% of patients received their first appropriate ICD therapy after battery replacement. In comparison, a previous study on patients with ICDs implanted for primary prevention reported that 27% received their first appropriate therapy after generator exchange (Madhavan et al. 2016). These findings highlight the difficulty of selecting patients who can avoid ICD implantation or generator exchange for secondary prevention, even in secondary prevention. However, alternative ICD options are available, including subcutaneous ICDs (S‐ICDs) and extravascular ICDs. For patients who do not require pacing for bradycardia, ventricular tachycardia termination, or cardiac resynchronization therapy (CRT), S‐ICD implantation is considered a Class IIa recommendation (Nogami et al. 2021). S‐ICDs are associated with a lower risk of major complications requiring intervention compared to transvenous ICDs (Olde Nordkamp et al. 2025). Therefore, for patients who are less likely to experience recurrent VT/VF, selecting S‐ICD may be a safer and more appropriate option. This underscores the importance of identifying individuals unlikely to experience future arrhythmic events.

### 4.2 Predictor of No Benefit From an ICD for Secondary Prevention

Lewenhardt et al. (2024) addressed a similar question by defining “no benefit from ICD implantation” as death from any cause without prior appropriate ICD therapy and analyzing the characteristics of such patients. Their findings showed that 20%–30% of patients across all age groups received appropriate ICD therapy, suggesting that elderly patients may still benefit from ICDs for secondary prevention. However, their study included only 42 patients who were followed until death or appropriate therapy, with a relatively short median follow‐up of 4.2 years. To more accurately identify predictors of no benefit from ICDs in secondary prevention, studies with larger cohorts and longer follow‐up periods—tracking patients until death or appropriate therapy—are needed.

In this study, older age and a history of VF were identified as predictors of no benefit from ICD implantation. Previous studies have also reported that a history of VT, rather than VF, is a stronger predictor of receiving appropriate ICD therapy (Schaer et al. 2016). A study on Japanese patients with ischemic heart disease also found that individuals with VT were significantly more likely to benefit from ICD therapy than those with VF (Hanada et al. 2025). Furthermore, prior reports have indicated that ICDs are less effective for secondary prevention in patients over 75 years of age (Healey et al. 2007). Appropriate ICD therapy typically occurs relatively early after implantation, as confirmed in this study with a median time of approximately 2 years. Therefore, the risk factors identified may have been consistent with the present studies (Schaer et al. 2016; Hanada et al. 2025). Additionally, trends show a decline in the rate of appropriate ICD therapy for secondary prevention between 2007 and 2016, suggesting a possible reduction in ICD efficacy for certain patient populations (Ruwald et al. 2021). Despite these trends, a notable proportion of elderly patients with a history of VF still receive appropriate ICD therapy (Figure 2). Although avoiding ICD implantation in this group remains challenging, the findings may aid in selecting the most suitable type of ICD for such patients.

### 4.3 Limitations

This study has several limitations that should be acknowledged. First, this was a retrospective, single‐center study with a relatively small sample size, which may have influenced the findings. Second, 37 patients currently under follow‐up at our hospital are still alive without having received appropriate ICD therapy. Therefore, their eventual outcomes could impact the study's conclusions and may introduce selection bias. However, in the Cox regression analysis of the 207 patients including the alive 37 patients without a history of appropriate ICD therapy, age > 70 years and a history of VF were similarly identified as independent predictors of death without appropriate ICD therapy during their lifetime (Table S1), and a history of VF was similarly identified as a predictor of freedom from appropriate ICD therapy (Table S2). Third, we classified patients who died within 1 year of receiving appropriate ICD therapy into the no‐benefit group; however, there is no established consensus on whether the 1‐year cutoff is appropriate. Notably, the results remained consistent even when only patients who died without any appropriate ICD therapy were included in the no‐benefit group (Table S3). Considering that only 30 patients died without receiving appropriate ICD therapy, larger‐scale prospective studies focusing on this patient population are required to validate and strengthen our findings.

## 5 Conclusion

Among patients who received a defibrillator for secondary prevention, 75% experienced a benefit through appropriate ICD therapy. However, the effectiveness of secondary prevention for VF appears to be limited in elderly patients. Therefore, careful consideration is warranted when selecting the type of defibrillator for this population.

## Author Contributions

All authors contributed to the study conception and design. Material preparation, data collection and analysis were performed by Sou Otsuki. The first draft of the manuscript was written by Rie Akagawa and all authors commented on previous versions of the manuscript. All authors read and approved the final manuscript. All authors take responsibility for all aspects of the reliability and freedom from bias of the data presented and their discussed interpretation.

## Funding

The authors have nothing to report.

## Disclosure

No generative artificial intelligence tools were used in the writing or editing of this manuscript.

## Ethics Statement

This study was approved by the Ethics committee of Niigata University and was conducted under the guidelines of the Declaration of Helsinki and in compliance with the Ethical Guidelines for Medical and Health Research Involving Human Subjects. Information concerning the implementation of the research, including the handling of existing specimens or information, and research implementation was made public, with opportunities provided for patients to withdraw from the study. Ethics committee of Niigata University considered that these activities met the criteria for ensuring patient consent and waived the need for written informed consent. We used the opt‐out method because of the retrospective nature of the study.

## Consent

Informed consent from all individual participants included in the study was waived by the ethics committee of Niigata University.

## Conflicts of Interest

The authors declare no conflicts of interest.

## Supporting information


**Table S1:** Cox regression analysis for prediction of death without a history of appropriate ICD therapy (including 37 alive patients without appropriate ICD therapy).
**Table S2:** Cox regression analysis for prediction of appropriate ICD therapy (including 37 alive patients without appropriate ICD therapy).
**Table S3:** Risk factors of death without appropriate ICD therapy (Among 167 patients).

## Data Availability

The data that support the findings of this study are available from the corresponding author upon reasonable request.
