# Supplementary material for: Benefits of Implantable Cardioverter–Defibrillator for Secondary Prevention in Patients With Organic Heart Disease
Source: Ann Noninvasive Electrocardiol. 2025 Dec 10;31(1):e70131. doi: 10.1111/anec.70131 (PMC12696040; doi:10.1111/anec.70131)
Supplement: Supplementary file 1 — Table S1: Cox regression analysis for prediction of death without a history of appropriate ICD therapy (including 37 alive patients without appropriate ICD therapy). Table S2: Cox regression analysis for prediction of appropriate ICD therapy (including 37 alive patients without appropriate ICD therapy). Table S3: Risk factors of death without appropriate ICD therapy (Among 167 patients). [file ANEC-31-e70131-s001.docx]

**Supplemental table 1. Cox regression analysis for prediction of death without a history of appropriate ICD therapy (including** **37 alive patients without appropriate ICD therapy)**

|  | **Univariate** | | | **Multivariate** | | |
| --- | --- | --- | --- | --- | --- | --- |
|  | OR | CI | *p* value | OR | CI | *p* value |
| Age >70 | 8.13 | 3.72-17.8 | **<0.001** | 4.46 | 1.67-11.9 | **0.003** |
| Female | 0.66 | 0.28-1.53 | 0.33 |  |  |  |
| BMI | 1.02 | 0.93-1.11 | 0.68 |  |  |  |
| Underlying disease |  |  |  |  |  |  |
| Ischemic cardiomyopathy | 4.72 | 2.25-9.90 | **<0.001** | 2.34 | 0.33-16.7 | 0.40 |
| Non-ischemic cardiomyopathy | 0.44 | 0.21-0.94 | **0.035** | 1.99 | 0.42-9.35 | 0.38 |
| Valvular heart disease | 0.63 | 0.09-4.63 | 0.65 |  |  |  |
| Congenital heart disease | 0.43 | 0.06-3.18 | 0.41 |  |  |  |
| CRT | 0.52 | 0.07-3.85 | 0.52 |  |  |  |
| VF episode | 2.65 | 1.29-5.45 | **0.008** | 2.83 | 1.33-6.01 | **0.007** |
| Comorbidities |  |  |  |  |  |  |
| Atrial fibrillation | 1.40 | 0.67-2.92 | 0.37 |  |  |  |
| Hypertension | 1.93 | 0.94-3.99 | 0.075 |  |  |  |
| Diabetes mellitus | 3.00 | 1.42-6.34 | **0.004** | 1.69 | 0.74-3.84 | 0.21 |
| Hyperlipidemia | 1.21 | 0.55-2.64 | 0.64 |  |  |  |
| eGFR<60 | 4.00 | 1.91-8.37 | **<0.001** | 1.87 | 0.79-4.46 | 0.16 |
| Anticoagulation | 1.78 | 0.79-4.00 | 0.16 |  |  |  |
| A history of malignant tumor | 1.21 | 0.37-4.02 | 0.75 |  |  |  |
| A history of PCI or CABG | 4.61 | 2.22-9.55 | **<0.001** | 1.36 | 0.33-5.61 | 0.67 |
| LV ejection fraction | 1.00 | 0.98-1.02 | 0.90 |  |  |  |
| QRS (/10ms) | 0.95 | 0.84-1.06 | 0.38 |  |  |  |
| QTc (/10ms) | 1.00 | 0.92-1.08 | 0.91 |  |  |  |
| Drug for heart failure |  |  |  |  |  |  |
| ACEi/ARB | 1.40 | 0.62-3.15 | 0.42 |  |  |  |
| MRA | 1.38 | 0.65-2.91 | 0.40 |  |  |  |
| β-blocker | 0.64 | 0.31-1.33 | 0.23 |  |  |  |
| Antiarrhythmic drug class III | 1.04 | 1.51-2.14 | 0.91 |  |  |  |
| Amiodarone | 1.69 | 0.70-4.09 | 0.24 |  |  |  |
| Sotalol | 0.45 | 0.17-1.17 | 0.10 |  |  |  |

Abbreviations are shown in Table 1.

**Supplemental table 2. Cox regression analysis for prediction of appropriate ICD therapy (including** **37 alive patients without appropriate ICD therapy)**

|  | **Univariate** | | | **Multivariate** | | |
| --- | --- | --- | --- | --- | --- | --- |
|  | OR | CI | *p* value | OR | CI | *p* value |
| Age >70 | 0.77 | 0.50-1.16 | 0.21 |  |  |  |
| Female | 0.66 | 0.44-0.99 | **0.044** | 0.57 | 0.38-0.87 | **0.009** |
| BMI | 1.01 | 0.97-1.06 | 0.60 |  |  |  |
| Underlying disease |  |  |  |  |  |  |
| Ischemic cardiomyopathy | 0.56 | 0.38-0.84 | **0.005** | 0.67 | 0.30-1.48 | 0.32 |
| Non-ischemic cardiomyopathy | 1.57 | 1.12-2.20 | **0.009** | 1.02 | 0.65-1.59 | 0.94 |
| Valvular heart disease | 0.62 | 0.25-1.51 | 0.29 |  |  |  |
| Congenital heart disease | 0.88 | 0.43-1.81 | 0.73 |  |  |  |
| Others | 1.52 | 0.90-2.56 | 0.12 |  |  |  |
| CRT | 1.32 | 0.76-2.30 | 0.32 |  |  |  |
| VF episode | 0.38 | 0.25-0.59 | **<0.001** | 0.43 | 0.28-0.67 | **<0.001** |
| Comorbidities |  |  |  |  |  |  |
| Atrial fibrillation | 0.84 | 0.59-1.22 | 0.36 |  |  |  |
| Hypertension | 0.90 | 0.63-1.28 | 0.54 |  |  |  |
| Diabetes mellitus | 0.95 | 0.62-1.45 | 0.82 |  |  |  |
| Hyperlipidemia | 0.82 | 0.56-1.20 | 0.30 |  |  |  |
| eGFR<60 | 0.99 | 0.69-1.41 | 0.94 |  |  |  |
| Anticoagulation | 1.06 | 0.75-1.49 | 0.76 |  |  |  |
| A history of malignant tumor | 0.71 | 0.35-1.46 | 0.36 |  |  |  |
| A history of PCI or CABG | 0.54 | 0.34-0.83 | **0.006** | 0.60 | 0.27-1.35 | 0.22 |
| LV ejection fraction | 0.98 | 0.97-0.99 | **0.007** | 0.98 | 0.97-0.99 | **0.001** |
| QRS (/10ms) | 1.03 | 0.98-1.08 | 0.20 |  |  |  |
| QTc (/10ms) | 0.99 | 0.95-1.03 | 0.77 |  |  |  |
| Drug for heart failure |  |  |  |  |  |  |
| ACEi/ARB | 0.80 | 0.55-1.15 | 0.22 |  |  |  |
| MRA | 1.03 | 0.72-1.47 | 0.87 |  |  |  |
| β-blocker | 1.23 | 0.83-1.84 | 0.29 |  |  |  |
| Antiarrhythmic drug class III | 1.51 | 1.07-2.14 | **0.019** |  |  |  |
| Amiodarone | 1.16 | 0.79-1.71 | 0.45 |  |  |  |
| Sotalol | 1.56 | 1.08-2.25 | **0.017** | 1.09 | 0.73-1.63 | 0.67 |

Abbreviations are shown in Table 1.

**Supplemental table 3. Risk factors of death without appropriate ICD therapy (Among 167 patients)**

|  | **Univariate** | | | **Multivariate** | | |
| --- | --- | --- | --- | --- | --- | --- |
|  | OR | CI | *p* value | OR | CI | *p* value |
| Age >70 | 4.88 | 2.12-11.2 | **<0.001** | 2.90 | 1.08-7.80 | **0.035** |
| Female | 1.04 | 0.41-2.65 | 0.93 |  |  |  |
| BMI | 1.02 | 0.92-1.12 | 0.75 |  |  |  |
| Underlying disease |  |  |  |  |  |  |
| Ischemic cardiomyopathy | 4.29 | 1.88-9.78 | **<0.001** | 1.13 | 0.16-8.23 | 0.90 |
| Non-ischemic cardiomyopathy | 0.40 | 0.17-0.92 | **0.031** | 1.01 | 0.23-4.41 | 0.99 |
| Valvular heart disease | 0.91 | 0.10-8.09 | 0.93 |  |  |  |
| Congenital heart disease | 0.56 | 0.07-4.62 | 0.59 |  |  |  |
| Others | 0.26 | 0.03-2.05 | 0.20 |  |  |  |
| CRT | 0.30 | 0.04-2.40 | 0.26 |  |  |  |
| VF episode | 4.07 | 1.78-9.35 | **<0.001** | 5.04 | 1.91-13.3 | **0.001** |
| Comorbidities |  |  |  |  |  |  |
| Atrial fibrillation | 1.46 | 0.65-3.29 | 0.36 |  |  |  |
| Hypertension | 1.57 | 0.71-3.49 | 0.27 |  |  |  |
| Diabetes mellitus | 2.25 | 0.96-5.28 | 0.061 |  |  |  |
| Hyperlipidemia | 1.25 | 0.52-2.98 | 0.62 |  |  |  |
| eGFR<60 | 2.90 | 1.29-6.54 | **0.010** | 2.51 | 0.94-6.74 | 0.067 |
| Anticoagulation | 1.68 | 0.70-4.05 | 0.25 |  |  |  |
| A history of malignant tumor | 1.79 | 0.45-7.19 | 0.41 |  |  |  |
| A history of PCI or CABG | 5.12 | 2.21-11.8 | **<0.001** | 3.09 | 0.59-16.3 | 0.18 |
| LV ejection fraction | 1.01 | 0.99-1.04 | 0.24 |  |  |  |
| QRS (/10ms) | 0.91 | 0.80-1.05 | 0.19 |  |  |  |
| QTc (/10ms) | 0.99 | 0.91-1.09 | 0.86 |  |  |  |
| Drug for heart failure |  |  |  |  |  |  |
| ACEi/ARB | 1.26 | 0.52-3.05 | 0.61 |  |  |  |
| MRA | 1.15 | 0.50-2.61 | 0.75 |  |  |  |
| β-blocker | 0.48 | 0.21-1.09 | 0.079 |  |  |  |
| Antiarrhythmic drug class III | 0.72 | 0.33-1.60 | 0.42 |  |  |  |
| Amiodarone | 0.89 | 0.35-2.25 | 0.80 |  |  |  |
| Sotalol | 0.45 | 0.16-1.26 | 0.13 |  |  |  |

Abbreviations are shown in Table 1.
